# Longer-term mortality following SARS-CoV-2 infection in people with severe mental illness: retrospective case-matched study

**DOI:** 10.1192/bjo.2021.1046

**Published:** 2021-11-02

**Authors:** Shanquan Chen, Emilio Fernandez-Egea, Peter B. Jones, Jonathan R. Lewis, Rudolf N. Cardinal

**Affiliations:** Department of Psychiatry, University of Cambridge, UK; Department of Psychiatry, University of Cambridge, UK; and Cambridgeshire and Peterborough NHS Foundation Trust, Fulbourn, UK; Department of Psychiatry, University of Cambridge, UK; and Cambridgeshire and Peterborough NHS Foundation Trust, Fulbourn, UK; and NIHR Applied Research Collaboration, East of England, UK; Cambridgeshire and Peterborough NHS Foundation Trust, Fulbourn, UK; Department of Psychiatry, University of Cambridge, UK; and Cambridgeshire and Peterborough NHS Foundation Trust, Fulbourn, UK

**Keywords:** Severe mental illness, SARS-CoV-2 infection, mortality, retrospective case-matched

## Abstract

Persisting symptoms and dysfunction after SARS-CoV-2 infection have frequently been observed. However, information on the aftermath of COVID-19 is inadequate. We followed up people with severe mental illness (SMI) infected with SARS-CoV-2, and evaluated their longer-term mortality, using data from Cambridgeshire and Peterborough NHS Foundation Trust, UK. We examined the time course and duration of mortality risk from the point of diagnosis. After SARS-CoV-2 infection, people with SMI had a substantially higher risk of death (hazard ratio (HR) = 5.16, 95% confidence interval (CI) 1.56–17.03; *P* = 0.007) during the first 28 days and during the following 28–60 days (HR = 2.96, 95% CI 1.21–7.26; *P* = 0.018) than those without infection, but after 60 days the additional risk of death was no longer significant (HR = 2.33, 95% CI 0.83–6.53; *P* = 0.107).

People with severe mental illness (SMI) are at increased risk of being infected by SARS-CoV-2 and have increased mortality and a more severe course of COVID-19.^1–5^ In the UK and other countries, the attribution of death to COVID-19 is restricted to the first 28 days after a positive COVID-19 test.^6^ Peak mortality is estimated to be 9–16 days after diagnosis.^7,8^ However, 45-day mortality in SMI is elevated after COVID-19 infection.^2^ The full time course and duration of elevated mortality risk following COVID-19 infection in SMI is therefore unclear, and one possibility is that the mortality risk extends beyond 28 days. In this study, we conducted a longer-term follow-up of this group of patients and explored their longer-term mortality. We hypothesised that the mortality caused by SARS-CoV-2 infection beyond 28 days is still significant.

## Method

### Study design and participants

We conducted a retrospective case-matched study using data from the mental health electronic clinical records of Cambridgeshire and Peterborough NHS Foundation Trust (CPFT) in the UK. CPFT covers a population of approximately 0.86 million people and provides secondary mental health and community physical health services. Data from CPFT include sociodemographic information, diagnosis, smoking status and death status. Clinicians enter this information during patients’ routine treatment in a systematic and standard way to ensure its accuracy.^9^ SARS-CoV-2 status was confirmed by the reverse transcription polymerase chain reaction (RT-PCR) test recommended by the World Health Organization (WHO).

Data were collected from 1 March 2020 to 28 February 2021, to avoid the influence of the SARS-CoV-2 vaccination widely available from March 2021 in the UK. Eligible patients were those diagnosed with SMI. SMI was judged on the basis of the ICD-10 diagnosis codes F20–F29 (including schizophrenia, delusional disorders and schizotypal disorder), F30 (manic episode) and F31 (bipolar affective disorder). The exposure cohort were people with SMI who had laboratory-confirmed SARS-CoV-2. The control group were matched (at a ratio of 1:10) from the remaining people with SMI, matched on age (±1 year) and gender. The origin time was the date of COVID-19 diagnosis (exposure cohort) or the matched exposed case's date of COVID-19 diagnosis (control cohort). Follow-up was until the study end date, the patient’s final recorded CPFT data or the date of their death, whichever occurred first.

### Data collection

The following sociodemographic variables were extracted: age at baseline (years), gender (female versus male), marital status (married, civil partnership or cohabiting versus not) and ethnicity (White versus other/not known). Smoking status was defined as being a past or current smoker. Death status for all patients known to the CPFT was extracted by weekly linkage to the national NHS Spine mortality data.

We selected comorbidities that were plausible risk factors for a worse prognosis^2,10^ as follows: myocardial infarction (ICD-10 codes I21–I25), hypertension (ICD-10 H35.03, I10–I16, I67.4, O10.1–O10.4 and O10.9), heart failure (ICD-10 I11.0, I13.0, I13.2, I42 and I50), diabetes mellitus (ICD-10 E08–E13), chronic kidney disease (ICD-10 D63.1, E08.22, E10.22, E11.22, E13.22, I12, I13, N03, N18, O10.3, Z49, Z91.15 and Z99.2), obstructive lung disease (ICD-10 J40–J47, J60, J66, J67.2, J67.8, J67.9, J68.4, J84 and G47.3) and cancer (ICD-10 C00–D49).

### Statistical analysis

Continuous variables were reported as mean (s.d.) and categorical variables were reported as *n* (%). Differences between groups were tested via two-tailed *t*-tests (for continuous variables) and chi-squared tests (for categorical variables).

**Fig. 1:**
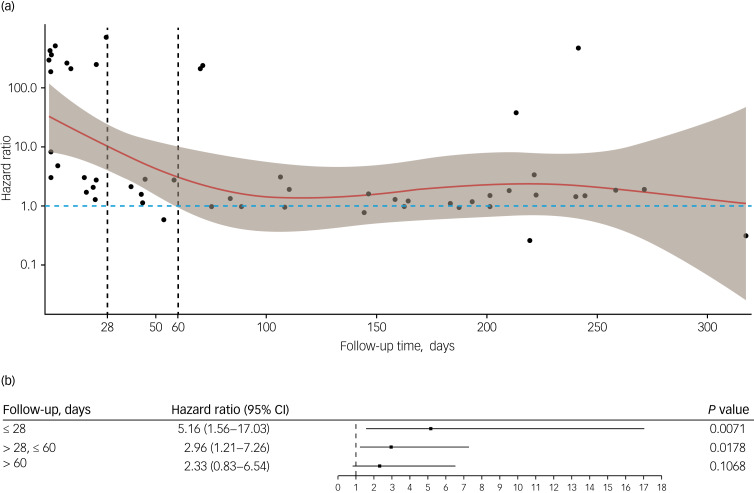
Association between SARS-CoV-2 infection and mortality, controlling for sociodemographic variables and comorbidities, with matching group identity as the cluster variable. (a) Hazard ratio (with 95% confidence interval (CI)) against follow-up time: the solid line is a natural spline fit (with 4 degrees of freedom as recommended^15^) of the time-varying estimates of the hazard ratio, with the shaded area representing a ±1.96 standard error band around the fit. (b) Piecewise results of the association between SARS-CoV-2 infection and mortality.

The Cox proportional hazard model was used to estimate the association between SARS-CoV-2 infection and mortality, controlling for sociodemographic variables and comorbidities, with matching group identity as a cluster variable. We first plotted the hazard ratio (HR) against follow-up time from the overall Cox regression, extracted from Schoenfeld residuals, a method originally used for graphically testing the proportional hazards assumption. We then conducted piecewise Cox regression to estimate the effect of SARS-CoV-2 infection on mortality during different follow-up periods. We chose 28 days and 60 days as the follow-up thresholds: 28 days is the limit of COVID-attributable death according to the National Health Service (NHS)^6^ and 60 days was derived from the plot (Fig. 1(a)).

All analyses were done in R (v3.5.0) on Windows. *P* < 0.05 was treated as statistical significance. We report results following the STROBE checklist.

### Ethics statement

The authors assert that all procedures contributing to this work comply with the ethical standards of the relevant national and institutional committees on human experimentation and with the Helsinki Declaration of 1975, as revised in 2008. All procedures involving human participants/patients were approved by an NHS Research Ethics committee (reference 17/EE/0442). Individual patient consent was not necessary as data were extracted from de-identified electronic clinical records; no patients were involved in the development of the research question or the outcome measures, or in developing plans for the design and analysis of the study.

## Results

Between 1 March 2020 and 28 February 2021, a total of 121 individuals with SMI and infected with SARS-CoV-2 were included in the exposure cohort, and 1210 individuals with SMI without SARS-CoV-2 infection were matched from 22 048 individuals with SMI and included in the control cohort (supplementary material available at https://doi.org/10.1192/bjo.2021.1046). The SARS-CoV-2 exposure cohort was followed up for an average of 159 days (s.d. = 123.6), significantly lower than that in the control cohort (186 days (s.d. = 120.1); *P* = 0.025). Some differences in baseline characteristics were identified between the two cohorts (see supplementary material). Individuals in the SARS-CoV-2 cohort were more likely to be of ‘other/not known’ ethnicity (*P* = 0.003), to be former/current smokers (*P* = 0.001), and to have comorbid hypertension (*P* = 0.004), diabetes mellitus (*P* = 0.016), chronic kidney disease (*P* < 0.001) or cancer (*P* = 0.029). No significant difference was observed between the two cohorts for other characteristics, including age, gender, marital status, heart failure, myocardial infarction or obstructive lung disease. Sixteen deaths (13.2%, or 303.6 per 1000 persons per year) were identified in the SARS-CoV-2 exposure cohort, a significantly higher fraction (*P* < 0.001) than in the unexposed cohort (33 deaths, 2.7%, or 53.5 per 1000 persons per year).

The Schoenfeld residuals from the overall Cox regression suggested that the effect of SARS-CoV-2 infection on mortality diminished from the start of the follow-up and disappeared from approximately 60 days until the end of the follow-up period (Fig. 1(a)). The piecewise regression indicated that individuals with SMI exposed to SARS-CoV-2 had a 4.16-fold higher risk of death (HR = 5.16, 95% CI 1.56–17.03; *P* = 0.007) during the first 28 days, and this additional death risk reduced to 1.96-fold but was still significant (HR = 2.96, 95% CI 1.21–7.26; *P* = 0.018) during the period of 28–60 follow-up days. After 60 days the additional risk of death following SARS-CoV-2 infection was not significant (HR = 2.33, 95% CI 0.83–6.54; *P* = 0.107) (Fig. 1(b)).

Results remained consistent after removing one patient with myocardial infarction and three patients with cancer.

## Discussion

These findings supported our hypothesis that the additional mortality following SARS-CoV-2 infection persists beyond 28 days, although it decreases over time, and also indicated that this increased risk for mortality was no longer statistically significant about 60 days after infection. Our results before the 28-day point are in line with previous studies, which concluded that people with SMI have an increased risk for COVID-19-associated mortality.^1,2,4,5,10,11^ Our study has quantified the dynamic change of this association and reports on the aftermath of COVID-19 in people with SMI in a longer follow-up period. Our results have implications for clinical practice and suggest that people with SMI who are infected with SARS-CoV-2 should have follow-up observations for at least 60 days even if they have recovered or been discharged. Our findings also suggest that the 28-day cut-off adopted by the UK^6^ might underestimate COVID-19-associated mortality in this population.

To the best of our knowledge, this is the first study to examine mortality in people with SMI following COVID-19 infection using uninfected people with SMI as a comparison group. This is a strength of our study, as people with SMI are at increased risk of premature mortality due to a variety of causes. The crude death rate in our control group is 53.5 per 1000 persons per year, higher than that reported in general populations (10.4 per 1000 persons per year in 2020).^12,13^ This study design provides a more accurate assessment of excess mortality related to COVID-19 infection in this population.

The current work has several limitations. First, our data did not contain information on the severity of symptoms for individuals infected with SARS-CoV-2. The long-term health sequelae are particularly relevant to those who required mechanical ventilation, for whom long-term complications and incomplete recovery would more likely be expected.^1,14^ Second, the sample is small, which prevented us from performing subgroup analyses for different types of SMI. Third, the control cohort may have included people with undiagnosed COVID-19 infection. Fourth, the use of secondary care data may have led to unmeasured biases (versus people with SMI not under the care of secondary mental health services). Fifth, the retrospective study design did not allow collection of data on persistent symptoms/disability among survivors of SARS-CoV-2 infection. Prospective studies in this area are required, considering also the specific cause of death. Similar studies in the general population are also needed, as a prolonged mortality risk may be a more widespread phenomenon.

## Data Availability

Patient-level data are not publicly available, under NHS research ethics terms. Source code and summary data are available from the corresponding author on request.

## Supplementary material

For supplementary material accompanying this paper visit http://dx.doi.org/10.1192/bjo.2021.1046. click here to view supplementary material
