# Supplementary material for: Longer-term mortality following SARS-CoV-2 infection in people with severe mental illness: retrospective case-matched study
Source: BJPsych Open. 2021 Nov 2;7(6):e201. doi: 10.1192/bjo.2021.1046 (PMC8564024; doi:10.1192/bjo.2021.1046)
Supplement: Supplementary file 1 [file bjosup.zip › S2056472421010462sup002.docx]

**STROBE diagram showing construction of the cohorts**

**Patient characteristics.** Data are shown as mean (SD) or number (percentage). P values for age and follow-up days were obtained by t test, and for others via Pearson's chi-square test.

|  | **Without SARS-CoV-2 infection group  (n = 1,210)** | **SARS-CoV-2 infected group  (n = 121)** | **p value** |
| --- | --- | --- | --- |
| **Age (years) at entry** | 47.49(21.43) | 47.89(21.62) | 0.845 |
| **Gender (Female)** | 470(38.8) | 47(38.8) | 1.000 |
| **Marital status (married, cohabiting or civil partnership)** | 307(25.4) | 32(26.4) | 0.881 |
| **Ethnicity (White, versus other/unknown)** | 1,119(92.5) | 102(84) | **0.003** |
| **Smoker (current or former)** | 69(5.7) | 17(14) | **0.001** |
| **Hypertension (yes)** | 266(22) | 41(33.9) | **0.004** |
| **Heart failure (yes)** | 0(0) | 0(0) | - |
| **Myocardial infarction (yes)** | 1(0.1) | 1(0.8) | 0.434 |
| **Diabetes mellitus (yes)** | 104(8.6) | 19(15.7) | **0.016** |
| **Chronic kidney disease (yes)** | 69(5.7) | 19(15.7) | **< 0.001** |
| **Obstructive lung disease (yes)** | 113(9.3) | 16(13.2) | 0.224 |
| **Cancer (yes)** | 5(0.4) | 3(2.5) | **0.029** |
| **Follow-up days** | 185.56(120.12) | 158.96(123.69) | **0.025** |
